# Is erenumab an efficient alternative for the prevention of episodic and chronic migraine in Spain? Results of a cost-effectiveness analysis

**DOI:** 10.1186/s10194-024-01747-w

**Published:** 2024-03-15

**Authors:** Patricia Pozo-Rosich, José Luis Poveda, Carlos Crespo, María Martínez, José Manuel Rodríguez, Pablo Irimia

**Affiliations:** 1 Neurology Department, Headache Unit, Valld’Hebron University Hospital, Ps. Vall d’Hebron 119-12, 08035 Barcelona, Spain; 2 Headache Research Group, Medicine Departament, VHIR, Universitat Autònoma de Barcelona, Barcelona, Spain; 3 Pharmacy Department, Hospital Universitari I Politècnic La Fe, Valencia, Spain; 4 Axentiva Solutions, Barcelona, Spain; 5 G.M. Statistics Department, Universidad de Barcelona, Barcelona, Spain; 6 Novartis, Barcelona, Spain; 7 Department of Neurology, Headache Unit, Clínica Universidad de Navarra, Pamplona, Spain

**Keywords:** Chronic migraine, Episodic migraine, Topiramate, Erenumab, Cost-effectiveness, Spain

## Abstract

**Background:**

The reimbursement of erenumab in Spain and other European countries is currently restricted because of the cost of this novel therapy to patients with migraine who have experienced previous failures to traditional preventive treatments. However, this reimbursement policy should be preferably based on cost-effectiveness studies, among other criteria. This study performed a cost-effectiveness analysis of erenumab versus topiramate for the prophylactic treatment of episodic migraine (EM) and versus placebo for chronic migraine (CM).

**Methods:**

A Markov model with a 10-year time horizon, from the perspective of the Spanish National Healthcare System, was constructed based on data from responder and non-responder patients. A responder was defined as having a minimum 50% reduction in the number of monthly migraine days (MMD). A hypothetical cohort of patients with EM with one or more prior preventive treatment failures and patients with CM with more than two treatment failures was considered. The effectiveness score was measured as an incremental cost per quality-adjusted life year (QALY) gained and cost per migraine day (MD) avoided. Data from clinical outcomes and patient characteristics were obtained from erenumab clinical trials (NCT02066415, STRIVE, ARISE, LIBERTY and HER-MES). Deterministic and probabilistic sensitivity analyses were performed to validate the robustness of the model.

**Results:**

After a 10-year follow-up, the estimated QALYs were 5.88 and 6.11 for patients with EM treated with topiramate and erenumab, respectively. Erenumab showed an incremental cost per patient of €4,420 vs topiramate. For CM patients, erenumab resulted in 0.756 QALYs gained vs placebo; and an incremental cost of €1,814. Patients treated with erenumab achieved reductions in MD for both EM and CM (172 and 568 MDs, respectively). The incremental cost per QALY gained with erenumab was below the Spanish threshold of €30,000/QALY for both health and societal perspectives (EM €19,122/QALY and CM €2,398/QALY).

**Conclusions:**

Erenumab is cost-effective versus topiramate as a preventive treatment for EM and versus placebo for patients with CM from the perspective of the Spanish National Health System.

**Supplementary Information:**

The online version contains supplementary material available at 10.1186/s10194-024-01747-w.

## Background

Migraine is a complex neurological disorder characterised by recurrent moderate or severe headache attacks lasting 4–72 h. Individuals with episodic migraine (EM) typically experience 0–14 monthly migraine days (MMD), whereas those with chronic migraine (CM) experience ≥ 15 MMD, with ≥ 8 MMD meeting criteria for migraine without aura and/or responding to migraine-specific treatment [1].

Migraine reduces physical and emotional health-related quality of life (HRQoL) among individuals suffering from the condition [1, 2], and serious or very serious disability is consequently reported in 51% and 89% of patients with EM or CM, respectively [3]. The impact of migraine worsens with increasing treatment failures and the degree of disability [3], and migraine is currently ranked as the second most disabling condition worldwide [4, 5]. Furthermore, migraine has a very high economic cost, estimated at ≥ 0.2% of gross domestic product globally [6–8]. This cost is primarily associated with lost work hours (absenteeism) and reduced productivity (presenteeism). The costs derived from the loss of workplace productivity in patients with migraine are estimated to account for ≥ 60% of the total costs associated with the disorder [1, 9–12].

Migraine preventive therapy aims to reduce the frequency of migraine attacks by at least 50% while decreasing their duration and severity, and restoring the ability of the patient to function [13]. Preventive treatments include oral prophylactics such as β-blockers (propranolol, metoprolol), tricyclic antidepressants (amitriptyline), calcium antagonists (flunarizine), neuromodulators (topiramate and sodium valproate) and candesartan. However, oral prophylaxis is only maintained after 6 months by approximately 30% of patients with either EM or CM [14, 15]. The main reasons for discontinuing preventive treatment are tolerability and lack of efficacy [14].

Monoclonal antibodies (mAbs) targeting the calcitonin gene-related peptide (CGRP) receptor (erenumab) or the circulating CGRP (eptinezumab, fremanezumab, galcanezumab) are new treatments for migraine prevention [16–18]. From a regulatory standpoint, CGRP drugs have been approved in Spain for the prophylaxis of migraine in adults with at least 4 MMD. However, their elevated cost has led several European countries, including Spain, to restrict reimbursement to patients with ≥ 8 MMD with at least three previous preventive treatment failures, one of these being onabotulinumtoxinA in the case of patients with CM [13, 19]. Nonetheless, the latest reviews of European guidelines [20] and the National Headache Foundation [21] recommend the use of anti-CGRP mAbs as a first-line treatment, considering their favourable tolerability and efficacy profiles, and improved adherence to treatment [22, 23], and erenumab is now a first-line therapy in Germany [24]. It should be noted that erenumab is the only anti-CGRP that has demonstrated proven efficacy against oral preventive treatments in randomised clinical trials [25, 26].

A systematic literature review of prophylactic migraine drugs identified various cost-effectiveness studies for erenumab in countries such as the USA, Sweden and Greece, and with different comparators [27]. Cost-effectiveness analyses should consider time horizons that capture all costs and effects of the interventions under evaluation, regardless of when they occur [22, 23]. Herein, the pathology was modelled using a Markov model with a time horizon of at least 10 years. Furthermore, the HRQoL using quality-adjusted life years (QALYs) was assessed in addition to duration of life. Finally, the efficiency of the use of erenumab in Spain was analysed for the first time.

Therefore, this study aimed to analyse the cost-effectiveness of erenumab versus topiramate in patients with EM and versus placebo in patients with CM in the context of Spain. The choice of comparators is justified based on the actual clinical practise for topiramate and the comparators used in the different pivotal studies for placebo [28–31].

## Methods

An economic assessment model was developed from a decision tree at 12 weeks combined with a Markov model of 12-week cycles, covering a 10-year time horizon. The model included hypothetical notional adult patients with an average age of 41 years, of whom 80.5% were women, who presented ≥ 4 migraine episodes per month, with one or more previous preventive treatment failures for EM, and three or more previous treatment failures for CM, one of which included onabotulinumtoxinA for CM treatment. The model was based on data from erenumab clinical trials (NCT02066415 [31], STRIVE, ARISE, LIBERTY [28–30] and HER-MES [26]) and adjusted to reimbursement conditions in Spain.

Populations of 1,000 notional patients each with EM or CM were assessed separately using topiramate and placebo as respective comparators, which were selected based on available clinical evidence from head-to-head comparison studies (NCT02066415 [31], STRIVE, ARISE, LIBERTY [28–30] and HER-MES [26]) and their reimbursement status within the Spanish Healthcare System. Topiramate is currently used as a preventive treatment for these patients in clinical practise in Spain and its comparison with erenumab was reported in the HER-MES study [26], whereas placebo was used as the control arm analysed in the main pivotal randomised control trials focusing on erenumab (NCT02066415 [31], STRIVE, ARISE and LIBERTY [28–30]). Erenumab and topiramate were compared among patients with EM with one or more previous treatment failures. For patients with CM, erenumab and placebo were compared after ≥ 3 failed treatments, with one of these treatments being onabotulinumtoxinA. For patients with EM and CM, prescription for symptomatic treatment was considered with triptans, aspirin, ibuprofen, ketoprofen, paracetamol, and paracetamol with codeine.

Outcomes for the cost-effectiveness analysis were incremental cost per QALY gained and incremental cost per migraine day (MD) avoided. The incremental cost-effectiveness ratio (ICER) was contrasted against the efficiency threshold per QALY in Spain more frequently described in the literature (€30,000/QALY) [32]. Although the primary analysis was performed from a healthcare system perspective, a sensitivity analysis performed from a societal perspective. The cost-effectiveness analysis followed international guidelines [33], and applied a discount rate of 3% for costs (year 2023) and effects [34].

### Model structure

**Fig. 1:**
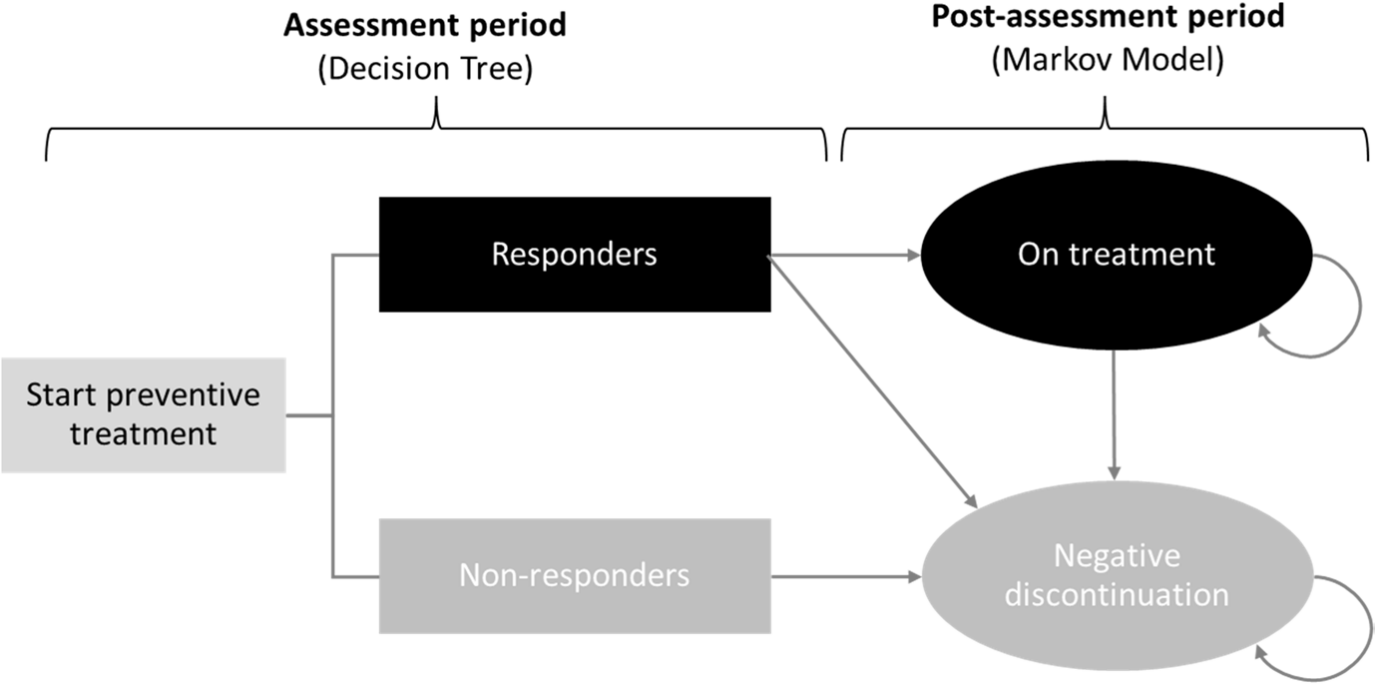
Model structure. All patients start preventive migraine treatment. Patients who at the end of 12 weeks have been classified as responders were assessed in the long term whether they remained on treatment or discontinued. Non-responders went directly to discontinuation status. Mortality was not considered as an individual health state, since this can occur to all patients, whatever their health status may be. Thus, the absorbing state for mortality is negative discontinuation

The course of the disorder was represented by a decision tree with two alternative branches (responders and non-responders), followed by a Markov model comprising two health states (treatment and discontinuation) with cycles lasting 12 weeks (Fig. 1). Model health states were exclusive, i.e. patients could only be in one specific health state at a specific time. Responders were defined as patients with 50% reduction in the number of MMD at week 12, regardless of whether they suffered from EM or CM.

All patients entered the model at treatment initiation and, after the assessment period (12 weeks), their response (i.e. responders or non-responders) to treatment was determined. Patients who responded (responders) could either continue the prophylactic treatment initiated (state: treatment) or discontinue if they had experienced adverse events (state: discontinuation). Patients in the treatment state were assumed to remain in that state until discontinuation due to adverse events. Costs and utilities were assigned based on the number of MMDs at the end of each cycle.

The mortality of the different states was considered similar to that of the general population, adjusted by age and sex, because no additional mortality was associated with migraine [35].

### Probabilities

Clinical data of the model were obtained from clinical trials of erenumab (Table 1). For the comparison between erenumab and topiramate in patients with EM, the HER-MES head-to-head study [26] was used. For the CM population, data for erenumab vs placebo were from Tepper et al. [31].

**Table 1:** Efficacy parameters used in the model

Comparators	Response	Odds ratio		Source
		**Mean**	**SE**	
Episodic migraine				
Erenumab + BSC	37%	-	-	Trial Data [28–30]
Topiramate + BSC	18%	2.76	1.16	HER-MES study [26]
Chronic migraine				
Erenumab + BSC	42%	-	-	Trial Data [31]
Placebo + BSC	17%	2.27	0.00	Trial Data [31]

*SE* Standard error, *BSC* best supportive care

Data available in the HER-MES study [26] and the NCT02066415 clinical trial [31] were used to recalculate the probabilities of the adverse events applied in the model, with adjustments period required for this study (12 weeks). Mean values were weighted based on the sample size of the available studies.

Following discontinuation due to adverse events, data included in the model were obtained from the main clinical trials with erenumab [26, 28, 31]. The topiramate discontinuation values were based on those described in the HER-MES study [26]. For EM, discontinuation probability per cycle was 5.52% for erenumab [26, 28, 31] and 35.57% for topiramate [26]. For CM, discontinuation for placebo was 0.71% and for erenumab was 1.06% [31].

Since the model considered a 10-year time horizon and the selected studies had only short terms, the probability of moving from treatment to discontinuation state after 12 weeks was estimated from the available long-term data and assumed for the 10-year time horizon. This produced a probability of long-term discontinuation for each cycle (12 weeks) of 30.26% (standard error [SE] 0.0177) for placebo [36], 5.1% (SE 0.0112) for topiramate after estimating the increase in discontinuation between weeks 12 and 24 of the HER-MES study [26] and 0.5% for erenumab based on the data of the 5-year extension study [37].

### Utilities

Utility refers to the value that an individual places on a health state: A score of 1 represents the best possible quality of life while a score of 0 represents a state equivalent to death. In this analysis, QALYs were calculated using estimated utilities obtained from the individual data collected from the trials [28, 31], by applying a linear regression based on MMD reduction, and considering the mortality of the general population. Consequently, utilities corresponding to each MMD state of the spectrum analysed (0–28 MMD) were calculated to give a mean reduction in utility per MMD of 0.0176 (SE 0.0035). Patients without migraine showed a utility of 0.85, patients with migraine and < 3 MMD showed a utility of 0.79–0.83, EM patients with < 7 MMD showed a utility of 0.72–0.78, EM patients with ≥ 8 MMD showed a utility of 0.6–0.71, CM patients with < 21 MMD showed a utility of 0.48–0.58, and CM patients with ≥ 21 MMD showed a utility of 0.36–0.46.

Based on the utility by MMD, a utility loss was applied for adverse events [28, 31, 38]. Utility losses applied to each treatment were based on frequency of onset as follows: brain fog -0.097 (SE 0.13), fatigue -0.061 (SE 0.097), exercise intolerance -0.048 (SE 0.092), insomnia -0.048 (SE 0.088), neck stiffness and pain -0.045 (SE 0.077), muscle weakness -0.034 (SE 0.058), sleepiness -0.03 (SE 0.059), constipation -0.029 (SE 0.06), drooping eyelids -0.024 (SE 0.067), respiratory tract infection -0.012 (SE 0.033), paraesthesia -0.012 (SE 0.045), dizziness -0.01 (SE 0.041), dry mouth -0.01 (SE 0.044), injection site pain -0.008 (SE 0.025) and itchiness -0.006 (SE 0.023) [38].

### Resource use and costs

Therapy costs were estimated using approved dosages (dose and frequency of use) and unit costs [39]. Ex-factory prices were used for hospital-dispensed treatments, and retail prices were applied for the remaining treatments, always using the reimbursed price. The corresponding discount according to Spanish Royal Decree 08/2010 [40] was applied.

For acute treatment with triptans, the pharmacological cost was estimated using the weighted average of almotriptan, eletriptan, frovatriptan, naratriptan, rizatriptan, sumatriptan and zolmitriptan, as well as the cost of the consumption of aspirin, ibuprofen, ketoprofen, paracetamol, and paracetamol with codeine.

The cost per health state, including hospitalisations, emergency room visits, appointments with specialists or primary care doctors, and concomitant medication (Table 2), was based on the average unit costs of each Spanish autonomous region [41–57] and the use of resources provided, which were validated by an expert panel comprising specialists in neurology, and hospital pharmacists with knowledge of the disorder. Hospitalisation costs were calculated considering the average cost of a complete stay due to migraine and other headaches (DRG 54) at the neurology unit of the 2020 Minimum Basic Database Set [58], which is an administrative database on hospitalised patients. All costs were inflated to 2023 costs using the Consumer Price Index [59].

**Table 2:** Resource use and costs by frequency of episode (12 weeks)

Resources for every 12 weeks	Unit cost	No migraine (0 MMD)		Low-frequency episode (1–3 MMD)		Intermediate-frequency episode (4–7 MMD)		High-frequency episode (8–14 MMD)		Chronic migraine (15 + MMD)	
		**Unit**	**% pat**	**Unit**	**% pat**	**Unit**	**% pat**	**Unit**	**% pat**	**Unit**	**% pat**
Hospitalisation	€2,044.61	-	-	1	5%	1	7%	1	7%	1	17%
Emergency visits	€227.70	-	-	1	29%	1	54%	1	74%	1	72%
Appointment with primary care doctor	€74.87	-	-	1	97%	-	-	-	-	-	-
Appointment with specialist (neurologist)	€104.36	-	-	1	4%	1	50%	1	100%	1	100%
Pharmacy visit (5 min)^a^	€5.89	-	-	-	-	1	100%	1	100%	1	100%
Triptans		€0.00		€0.80–€3.80		€5.30–€9.70		€11.20–€20.20		€21.70–€41.10	
Other medication		€3.56		€6.20–€8.20		€6.20–€8.20		€8.90–€12.90		€13.50–€22.20	

*MMD* Monthly migraine days

^a^Only applicable to hospital-dispensed drugs

For the societal perspective analysis, which corresponds to an alternative scenario, indirect costs, such as absenteeism and presenteeism, were included in the model. Out-of-pocket expenses were not considered as evidence is lacking for this in the field of migraine. The relationship between absenteeism measured by the MIDAS scale [60] and MMD was determined through a regression model, and the same was performed for presenteeism. After determining the volume, the cost of one day of lost work for patients with absenteeism was applied, along with half a day of work for patients with presenteeism [61].

To estimate the daily labour cost, the total monthly wage cost (€2,847.10) [62] was adjusted to 22 working days, considering an unemployment rate of 12.7% [63]. In the sensitivity analysis, the valuation of indirect costs was incorporated using WPAI [64] methodology.

### Sensitivity analysis

These models are inevitably subject to uncertainty, thus we conducted both deterministic and probabilistic sensitivity analyses, following the recommended guidelines [34]. To evaluate the potential impact of each assigned variable, a univariate analysis was performed, varying the parameters by 20%. The results are presented in a tornado diagram [65]. A univariate sensitivity analysis was also performed using different scenarios (number of treatment failures, absence of prior failure to onabotulinumtoxinA, societal perspective, measurement of absenteeism/presentism). The model was complemented with a probabilistic sensitivity analysis by applying lognormal distributions for odds ratio, beta distributions for probabilities and utility loss, multivariate normal distributions for utilities and for the regressions assessed, and gamma distributions for resource use and discontinuation rates [66]. The probabilistic sensitivity analysis was supported by a 1000-iteration Monte Carlo simulation, which draws random values of the parameters per iteration to provide a theoretical probability distribution. The probabilistic sensitivity analysis results were plotted into a cost-effectiveness acceptability curve [67].

## Results

### Episodic migraine

At baseline, patients with EM had an average MMD of 9.44, which decreased to 6.95, 7.23 and 7.42 at 12, 24 and 108 weeks (first two years), respectively, following treatment with erenumab. Patients with EM treated with topiramate had 8.56, 8.65 and 8.86 MMD on average at 12, 24 and 108 weeks. Thus, at 10 years, patients with erenumab would have experienced 877 MDs, while patients with topiramate would have experienced 1,049 MDs (Table 3).

**Table 3:** Cost-effectiveness results at 10-year follow-up

Comparators	Cost	Effectiveness		ICER
		MD	QALYs	
Episodic migraine				
Topiramate + BSC	€17,059	1,049	5.8839	-
Erenumab + BSC	€21,479	877	6.1150	€26/MD avoided
				€19,122/QALY gained
Chronic migraine				
Placebo + BSC	€24,921	2,100	4.5016	-
Erenumab + BSC	€26,734	1,532	5.2578	€3/MMD avoided
				€2,398/QALY gained

*BSC* best supportive care, *ICER* incremental cost-effectiveness ratio, *MD* migraine days, *QALYs* quality-adjusted life years

Considering both MD and HRQoL, erenumab displayed an increase in QALYs with respect to topiramate (0.2311 QALYs).

Healthcare costs reached €21,479 and €17,059 for erenumab and topiramate, respectively, and hospitalization accounted for 34% and 25% of their respective overall cost. In addition, the costs per emergency visit were 33% for topiramate and 24% for erenumab. For erenumab, the highest costs came from pharmacological treatment (29%). Considering the societal perspective, the 10-year costs of topiramate amounted to €53,157, whereas those of erenumab amounted to €52,393. The cost reduction with erenumab compared with other alternatives was mainly derived from the costs of absenteeism, which amounted to €13,934 and €16,396 for erenumab and topiramate, respectively. This difference increased upon inclusion of the costs of presenteeism (erenumab €16,979; topiramate €19,703). Thus, indirect costs constituted 59% and 68% of the costs of erenumab and topiramate, respectively.

**Fig. 2:**
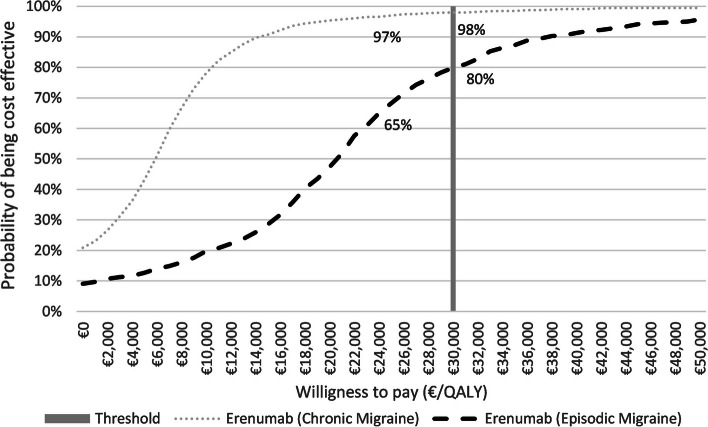
Willingness-to-pay curve. The acceptability curve allows the identification of the likelihood of erenumab being cost-effective against topiramate (dark black dashes) and against placebo (grey dots) according to different willingness to pay. The probability of erenumab being cost-effective is above > 50% in the potential efficiency threshold ranges in Spain (30,000€ per QALY)

For the costs and effectiveness at 10 years, the ICER between erenumab and topiramate in the Spanish context was €19,122 per QALY gained and €26 per MD avoided. The loss of quality of life based on MD and the probability of a response had the largest effect on the results based on the univariate sensitivity analysis (Supplementary Material S1). These results were confirmed by the probabilistic sensitivity analysis, indicating a probability of > 50% that erenumab is cost-effective for thresholds > €20,700 compared with topiramate (Fig. 2). From a societal perspective, erenumab would be the dominant alternative versus topiramate because it was more effective in reducing MMD and increasing QALY, with lower associated costs (Supplementary Material S2).

### Chronic migraine

Over the 10-year time horizon, patients with CM treated with erenumab showed a reduction of 568 MMD compared with that of placebo. Hence, at 10 years, patients with a baseline mean MMD of 18.66 would reach a mean MMD of 18.32 with placebo and 13.48 with erenumab. These results translated to 4.5016 and 5.2578 QALYs for placebo and erenumab, respectively (Table 3).

Healthcare costs reached 26,734€ for erenumab and €24,921 for placebo. Hospital stays accounted for 44% of the costs of placebo and 32% of those of erenumab, while the costs per emergency visit were 24% for placebo and 21% for erenumab. Pharmacological treatment represented 22% of the total medical costs for erenumab. Considering the societal perspective, indirect costs were 73% of the total 10-year placebo cost (total costs €92,023) and 65% of erenumab cost (total costs €76,875). This was due to the high costs of presenteeism (€27,079 for erenumab and €35,989 for placebo) and absenteeism (€23,062 for erenumab and €31,113 for placebo).

The ICER between erenumab and placebo in the Spanish context resulted in 2,398€/QALY gained and €3 per MD avoided. From a societal perspective, which added productivity losses to the healthcare system perspective, the use of erenumab would be the dominant therapeutic strategy for patients with multiple preventive treatment failures, as this produced savings of €15,148 and improved effectiveness, measured both in QALYs gained and MD avoided (Supplementary Material S2).

Univariate sensitivity analysis from the healthcare perspective demonstrated that hospital costs, loss of quality of life based on MMD and probability of response to treatments were the greatest influence on the outcome (Supplementary Material S3). However, the ICER was not sufficiently modified in any of the cases to change the conclusion of the analysis. The robustness of the results was confirmed in the probabilistic sensitivity analysis, where the acceptability curve showed that erenumab becomes the option of choice when the willingness to pay per QALY gained exceeds €5,800, a figure well below the €30,000/QALY threshold commonly accepted in Spain [32] (Fig. 2). Scenario analysis showed that erenumab was cost-effective versus placebo, regardless of the number of prior treatment failures or whether patients had an absence of prior failure to onabotulinumtoxinA, with an ICER below €5,000/QALY gained (Supplementary Material S4).

## Discussion

Herein, economic data supported the evidence in favour of using erenumab to treat both CM and EM in terms of cost-effectiveness with a reduction of approximately 10% in direct nonpharmacological costs. Treatment with erenumab reduced costs at a societal level, which is especially relevant in migraine, where productivity loss constitutes 60–70% of total costs [1, 13]. Consequently, the erenumab-attributed reduction in MD can help prevent absenteeism and presenteeism [10, 12, 14]. The consideration of these costs under the societal perspective analysis identified erenumab as the dominant alternative, that is more effective and less costly than its comparators. The estimate of indirect costs followed a conservative approach, because other costs derived from caregivers or other indirect costs assumed by patients, such as out-of-pocket expenses, were not considered. Therefore, the differences in indirect costs observed in our study may have been underestimated. Our results are in line with findings from other countries [68–70], which have also highlighted the substantial socioeconomic gains associated with treatment with CGRP-mAbs.

However, despite the cost-effective profile in comparison with other treatments compared, the reimbursement of erenumab by the Spanish Health System is more restricted than the marketing authorisation provided by the European Medicines Agency (EMA) [19]. In Spain, erenumab is only reimbursed for patients who suffer ≥ 8 MMD and have had at least three previous treatment failures, whereas the EMA indication is the preventive treatment for migraine patients with at least 4 MMD. Our study is consistent with previous efficacy studies [27] and expands the comparisons available and the populations assessed. Specifically, our study demonstrated that erenumab is cost-effective compared with the use of best supportive care in the reimbursement conditions of Spain for treating patients with CM, and indicates that erenumab is cost-effective in treating patients with EM compared with an active comparator, even in earlier lines of therapy, according to its indication and its reimbursement in other countries [71]. Importantly, the best supportive care option may not be feasible in clinical practise, and assuming efficacy is difficult over a 10-year period.

At least 38% of patients who suffer from EM and all patients with CM would benefit from prophylactic therapy, although an estimated < 13% receive this [72]. The use of preventive therapy is decisive in improving HRQoL for patients with migraine, in addition to preventing progression to CM [13, 72].

Notably, classic preventive treatments may have certain limitations. For instance, oral treatments require titration to optimal doses and daily administration, with efficacy not observed until 4 weeks after initiation [73]. In addition, the main problems of traditional oral treatments are their poor adherence, persistence, and therapeutic compliance, primarily due to their modest effectiveness and limited tolerability [14, 36]. Conversely, erenumab is a subcutaneously administered drug with a monthly frequency [74] and the induced effect can be perceived by the patient within the first week after injection [75]. The HER-MES study results revealed that erenumab exhibited a more favourable tolerability and efficacy profile than topiramate, causing fewer discontinuations throughout the 24-week treatment period [14]. Based on these findings, recent guidelines recommend mAbs targeting the CGRP pathway as the first-line treatment for migraine prevention, with erenumab preferred over topiramate [24].

Erenumab is a new option for prophylactically treating migraine and demonstrates superior efficacy over the alternatives introduced in our study [26, 31]. The gain in QALYs is derived from MMD reduction, both for CM and EM. In addition to greater efficacy, erenumab exhibits an improved adherence profile, possibly associated with a monthly self-administration pattern [74], a faster onset of response [75], and higher tolerability than oral preventives [76]. Erenumab is the only anti-CGRP pathway mAb that has demonstrated superiority over oral preventives in head-to-head trials, not only against topiramate in the HER-MES [26] used for our analysis, but also in the recent APPRAISE trial [25] that demonstrated the sustained benefit of erenumab and superior persistence rate in patients with EM over oral preventives.

Our study has the inherent limitations of all economic assessment models based on literature [77]. However, conservative assumptions were adopted, based on which, a broad set of analytical scenarios was conducted. Lack of evidence regarding adherence or out-of-pocket expenses is a limitation that produces a conservative approach in the estimations. Should new evidence be published, an update of the model would be paramount to evaluate the impact of these variables on the cost-effectiveness of erenumab. Furthermore, the HER-MES study [26] was conducted in a different healthcare system than the Spanish one, where the patient profile comprises subjects in earlier stages of the disease and less refractory to treatments, which may differ from the notional patient specified by the reimbursement conditions in Spain [37, 74]. Therefore, more clinical evidence in line with Spanish reimbursement conditions is required to conduct more robust local economic assessments [78]. Similarly, future studies should incorporate real clinical practise data.

## Conclusions

Erenumab was found to be a cost-effective alternative for the prevention of EM and CM in Spain. The greater effectiveness of erenumab and the reduction of nonpharmacological healthcare costs and societal costs at 10 years make this a cost-effective option for migraine prophylaxis versus placebo in patients with CM who have failed three or more previous preventive treatments, and versus topiramate in patients with EM who have failed one or more preventive treatments, considering a willingness-to-pay threshold of €30,000/QALY [32]. Consequently, erenumab would benefit patients with migraine in the Spanish Healthcare System, even in earlier lines of therapy, as recommended by the latest European guidelines [20].

### Supplementary Information

**Supplementary Material 1. **

## Data Availability

No datasets were generated or analysed during the current study.

## Abbreviations

CGRP: Calcitonin gene-related peptide
CM: Chronic migraine
EM: Episodic migraine
EMA: European Medicines Agency
HRQoL: Health-related quality of life
ICER: Incremental cost-effectiveness ratio
mAbs: Monoclonal antibodies
MMD: Monthly migraine days
MD: Migraine days
QALY: Quality-adjusted life years
SE: Standard error
