# Supplementary material for: Is erenumab an efficient alternative for the prevention of episodic and chronic migraine in Spain? Results of a cost-effectiveness analysis
Source: J Headache Pain. 2024 Mar 15;25(1):40. doi: 10.1186/s10194-024-01747-w (PMC10943917; doi:10.1186/s10194-024-01747-w)
Supplement: Supplementary file 1 — Supplementary Material 1. [file 10194_2024_1747_MOESM1_ESM.pdf]

SUPPLEMENTARY MATERIAL

S1. Tornado diagram. Episodic Migraine

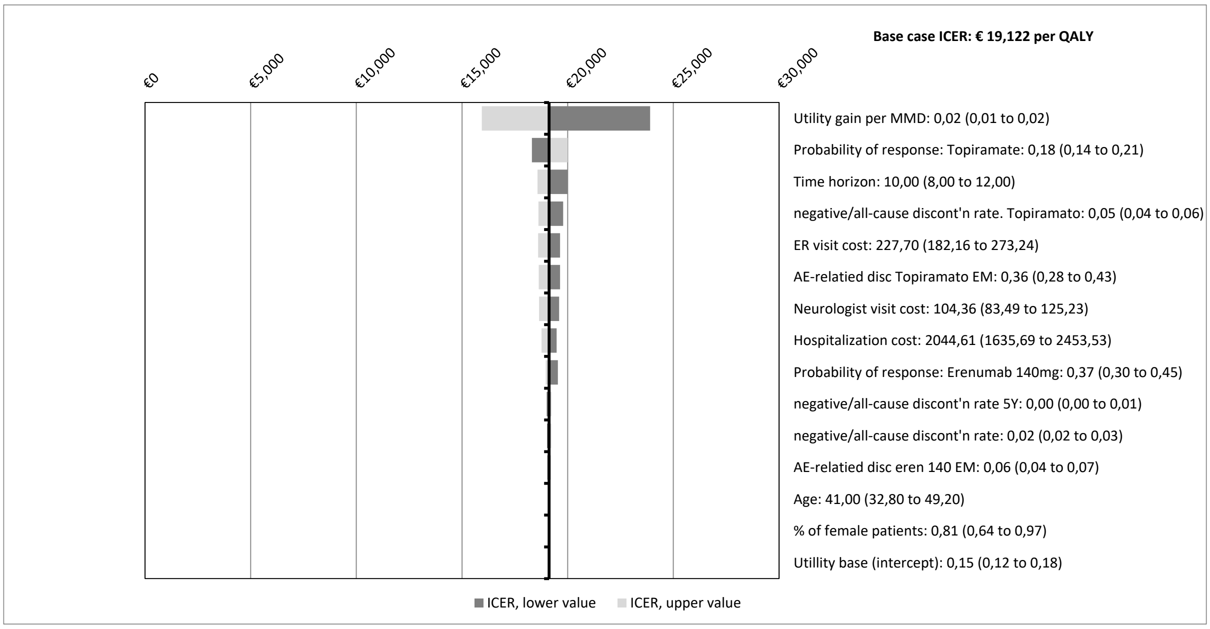

S2. Cost-effectiveness result at 10 years. Societal perspective

COMPARATORS	Cost	Effectiveness		ICER
		MD	QALYs	
Episodic Migraine				
Topiramate + BSC	€53,157	1,049	5.8839	-
Erenumab + BSC	€52,393	877	6.1150	Erenumab is dominant alternative
Chronic Migraine				
Placebo + BSC	€92,023	2,100	4.5016	-
Erenumab + BSC	€76,875	1,532	5.2578	Erenumab is dominant alternative

BSC: Best Supportive Care; ICER: Incremental Cost-effectiveness Ratio; MD: migraine days; QALYs: quality-adjusted life year

A dominant treatment option is one that is both less costly and results in better health outcomes than the comparator treatment.

S3. Tornado diagram. Chronic Migraine

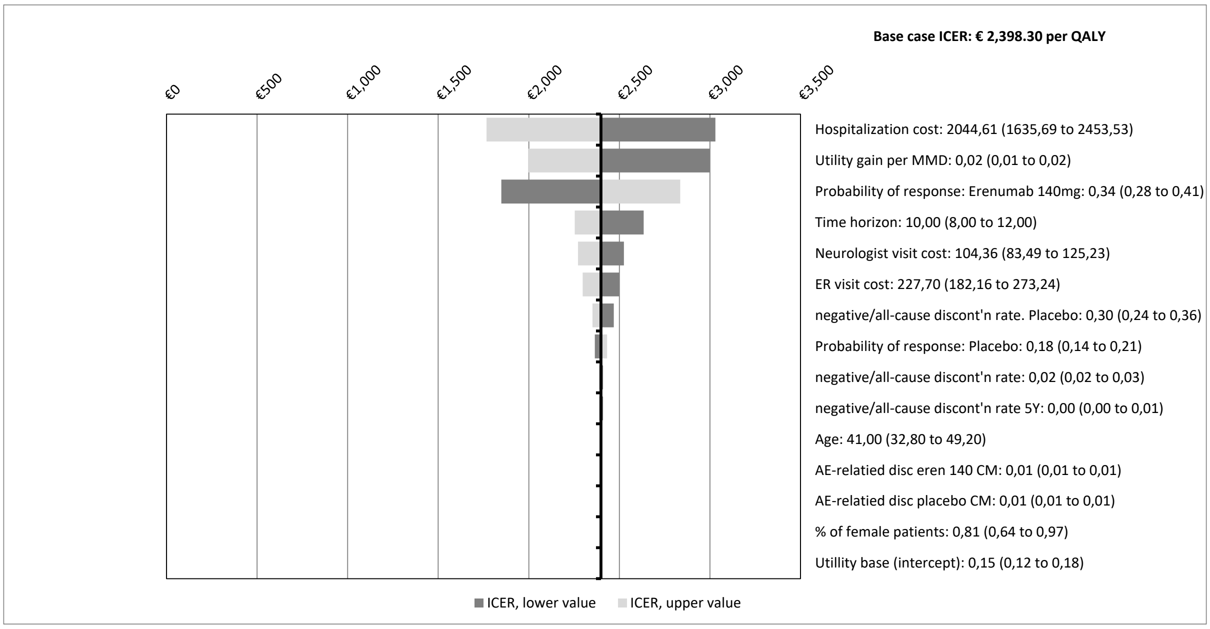

S4. Cost-effectiveness result. Scenario analysis

	ICER (€/MDD)		ICER (€/QALY)	
**BASE CASE**	**Episodic Migraine**	**Chronic Migraine**	**Episodic Migraine**	**Chronic Migraine**
BASE CASE	€26/MD	€3/MD	€19,122/QALY	€2,398/QALY
NUMBER OF PRIOR TREATMENT FAILURES				
1	€26/MD	€4/MD	€19,122/QALY	€3,291/QALY
2	€24/MD	€3/MD	€18,213/QALY	€2,595/QALY
3	€22/MD	€3/MD	€16,361/QALY	€2,398/QALY
FREQUENCY EPISODES				
Lower frequency episode	€47/MD	-	€34,432/QALY	-
All patients	€27/MD	-	€20,453/QALY	-
FAILURE TO ONABOTULINUMTOXINA				
Absence	-	€6/MD	-	€4,749/QALY
MEDICATION OVERUSE				
No	-	€6/MD	-	€4,365/QALY
DISCOUNT RATE FOR COST AND EFFECTS				
0%	€25/MD	€3/MD	€18,77/QALY	€2,279/QALY
1%	€25/MD	€3/MD	€18,891/QALY	€2,318/QALY
5%	€26/MD	€3/MD	€19,356/QALY	€2,480/QALY
TIME HORIZON				
3 years	€33/MD	€6/MD	€25,588/QALY	€4,746/QALY
5 years	€30/MD	€4/MD	€22,218/QALY	€3,321/QALY
15 years	€24/MD	€3/MD	€17,965/QALY	€2,086/QALY
30 years	€22/MD	€2/MD	€16,868/QALY	€1,677/QALY
AGE OF PATIENTS				
20 years old	€26/MD	€3/MD	€19,110/QALY	€2,394/QALY
60 years old	€25/MD	€3/MD	€19,207/QALY	€2,427/QALY
80 years old	€21/MD	€3/MD	€20,096/QALY	€2,721/QALY
PERCENTAGE OF FEMALE PATIENTS				
0%	€26/MD	€3/MD	€19,130/QALY	€2,401/QALY
100%	€26/MD	€3/MD	€19,120/QALY	€2,398/QALY
REIMBURSED PRICE				
No	€78/MD	€18/MD	€57,718/QALY	€13,799/QALY

ICER: Incremental Cost-effectiveness Ratio; MD: migraine days; QALYs: quality-adjusted life year
